# Impella 5.5 for Preoperative Optimization in the Setting of Ruptured Papillary Muscle

**DOI:** 10.1055/a-2603-9367

**Published:** 2025-05-30

**Authors:** David Rekhtman, Amit Iyengar, Jason Han, Rohan Shad, Marisa Cevasco, Wilson Szeto, Michael Ibrahim

**Affiliations:** 1 Division of Cardiovascular Surgery, Hospital of the University of Pennsylvania, Philadelphia, Pennsylvania, United States

**Keywords:** circulatory-assist devices, myocardial infarction, heart failure, mitral valve surgery, myocardial infarction

## Abstract

Cardiogenic shock in the setting of a ruptured papillary muscle can be extremely challenging to manage. We report two cases of successful and safe hemodynamic optimization with a modern axial-flow temporary left ventricular assist device, the Impella 5.5, prior to mitral valve replacement for this indication.

## Introduction

A ruptured papillary muscle precipitates cardiogenic shock requiring immediate surgical intervention. Temporary mechanical circulatory support (tMCS) has increasingly been used as a bridge to cardiac surgery, which allows the team to safely stabilize the patient prior to surgery, reduce perioperative mortality, and improve postoperative recovery. 1 2 The Impella 5.5 (Abiomed, Danvers, Massachusetts, United States) is the latest axial-flow pump device that increases antegrade flow resulting in decreased mitral regurgitation (MR) and pulmonary congestion. Device support has been described in patients after post-myocardial infarction (MI) mechanical complications. 3 4 However, use of the Impella 5.5 in the setting of a ruptured papillary muscle has not been reported.

We describe the successful perioperative use of Impella 5.5 for hemodynamic optimization of two patients in cardiogenic shock after post-MI papillary muscle rupture.

## Case 1

A 62-year-old female (body surface area [BSA]: 1.46 m ^2^ ) presented with dyspnea, lightheadedness, and syncope. She was found to have ST elevations in the inferior leads, severe MR, and a left ventricular ejection fraction (LVEF) of 35%. Given advanced shock, she was transferred to our center for urgent tMCS.

The patient was taken to the operating room (OR) for placement of an Impella 5.5. On intraoperative transesophageal echocardiogram (TEE), a ruptured posterior papillary muscle was noted. The decision was made to continue with implantation using the right axillary artery and an 8-mm Gelweave graft (Terumo Aortic, Somerset, New Jersey, United States). The patient left the OR with a mean arterial pressure of 70 mm Hg on moderate inotropic support; the Impella was set to P6 with a flow of 3.8L/min. In the intensive care unit (ICU), the cardiac index improved, urine output increased, and lactate cleared. After resolution of presenting shock, improvement in end-organ function, successful diuresis, and completion of goals-of-care discussions, the patient was felt to be an acceptable operative candidate.

On postoperative day (POD) 2, the patient returned to the OR for a mitral valve replacement (MVR). A standard sternotomy was performed. The mitral valve was exposed through a left atriotomy and replaced with a 25-mm St. Jude Epic (Abbott, Chicago, Illinois, United States) bioprosthetic valve. Postoperative TEE revealed functioning valves with an LVEF of 35%. The Impella was kept in place for postcardiotomy support and set to P4 with a flow of 1.7 L/min. The patient was extubated the following morning. Once perioperative diuresis was achieved and the patient appeared clinically stable, Impella support was decreased while chemical and vasopressor support was titrated to maintain a cardiac index > 2.2 L/min/m ^2^ . The patient was maintained on minimal mechanical support to prove sufficient cardiac function prior to Impella removal (POD 6).

Her postoperative course was complicated by pneumonia and subsequent respiratory failure requiring reintubation and tracheostomy on POD 13. Ultimately, the patient was discharged to a skilled-nursing facility where she stayed for an additional week before returning home. She is now doing well.

## Case 2

**Supplementary Video S1** Intraoperative TEE of ruptured papillary muscle. TEE, transesophageal echocardiogram.


**Supplementary Video S2** Intraoperative TEE after Impella placement. TEE, transesophageal echocardiogram.


A 65-year-old male (BSA: 2.22 m ^2^ ) presented to an outside hospital with dyspnea and radiographic pulmonary congestion. He was initially diagnosed with pneumonia and was placed on veno-venous extracorporeal membrane oxygenation (VV-ECMO) for profound hypoxic failure. Further workup revealed a subacute inferior STEMI and cardiogenic shock. Thus, the patient was transferred directly to the OR for Impella 5.5 placement. Intraoperative TEE showed posterior mitral valve papillary muscle rupture with torrential MR ( Supplementary Videos S1 , S2 ). The Impella was placed and set to P8 with 3.9 L/min of flow and VV-ECMO flow was set to 4.8 L/min. In the ICU, the patient remained on moderate doses of pressors while showing signs of improvement in cardiac index (2.23 L/min/m ^2^ ) and clearance of lactate from 6 to 1.1 mmol/L.

Following initial clinical stability, elevated pulmonary artery pressures and new right heart dysfunction were noted. It was felt, given his size, the Impella offered inadequate forward flow for full right-sided decompression. As such, veno-arterial-venous ECMO was initiated and the Impella was transitioned to use as a vent on POD 5. The patient was taken to the OR on POD 7 for an MVR. In standard fashion, a 33-mm St. Jude Epic valve was implanted. The postoperative LVEF was 25 to 30% with right ventricle function suitable for arterial decannulation. The patient returned to the ICU on VV-ECMO and Impella at P8 with 4.8 L/min of flow. The patient continued to improve with a significant reduction in pulmonary congestion and a return of native cardiac function noted on subsequent days.

He underwent tracheostomy on POD 21 for prolonged ventilator-dependent respiratory failure. He was successfully decannulated from VV-ECMO on POD 24, and the Impella was subsequently removed on POD 26.

The patient developed pseudomonal pneumonia and candida fungemia on POD 29. Despite prolonged antimicrobial and vasopressor support, the patient progressed to refractory septic shock. Ultimately, the family requested transition to comfort care, and the patient expired.

## Discussion

MI predisposes the structures supplied by the corresponding coronary vasculature to further damage in the recovery period. Rupture of the papillary muscle is a rare mechanical complication that can lead to torrential MR with associated pulmonary edema and shock. 5 It is essential to recognize and intervene to prevent rapid clinical deterioration and death.

The management of a post-MI complication is challenging given the complex presentation of cardiogenic shock and severe MR. Thus, a strategy that can offload the LV, decongest the lungs, and provide sufficient cardiac output is essential. An axial-flow pump can achieve these clinical goals. The theoretical risk of device thrombosis or embolic dislocation of debris has not previously been reported. Both patients in this study were maintained on a heparin drip with a partial thromboplastin time goal of 40 to 50 seconds as well as the standard Impella purge solution. Consistent with existing literature, no device complications or neurologic deficiencies were seen while on Impella support. 6 Of note, previous models with longer motor units have caused papillary rupture and mitral valve damage, but this has not been reported in the Impella 5.5 literature. 7 Therefore, surgical teams should consider isolated Impella as an option for these patients.

ECMO has been the standard for acutely increasing cardiac output and may have a role in acute shock following an MI. However, ECMO increases LV afterload resulting in an increase in LV end-diastolic pressure and associated left atrial dilation and pulmonary edema. 8 In the setting of torrential MR, these complications are particularly harmful requiring additional LV unloading. Concomitant use of the Impella with ECMO (ECPella) has been used to mitigate these ECMO-related complications by increasing forward flow. 3 The quality of support provided by the Impella may depend on the patient's BSA with the device capturing sufficient cardiac output in smaller patients. 9 In our report, the patient with a BSA of <2.0 m ^2^ was only supported with an Impella, whereas the patient with a BSA of >2.0 m ^2^ required additional ECMO support. Additionally, the Impella, being designed to offload the LV, may not be sufficient in the setting of biventricular failure. Although the ECPella can provide significant benefit through hemodynamic stabilization, ECMO use, and its associated complications, may be avoided by utilizing the Impella in certain patient populations.

In this case, we report the successful use of Impella 5.5 for preoperative optimization for MVR in the setting of a ruptured papillary muscle. We discussed the pathophysiology of post-MI complications as well as the considerations of utilizing the ECPella model. These cases emphasize the multipurpose use of novel technologies to benefit patients with varied underlying pathologies. In conclusion, ruptured papillary muscles should not be seen as a contraindication for axial-flow pump devices.

## References

[1] Benke K Korça E Boltjes A Preventive Impella ^®^ support in high-risk patients undergoing cardiac surgery J Clin Med 2022 11 18 5404 36143050 10.3390/jcm11185404 PMC9504963

[2] Pieri M D'Andria Ursoleo J Nardelli P Temporary mechanical circulatory support with Impella in cardiac surgery: a systematic review Int J Cardiol 2024 396 131418 37813286 10.1016/j.ijcard.2023.131418

[3] Gregory V Grunfeld M Kanwal A Escalation from Impella 5.5 to ECPELLA support as a bridge to mitral valve surgery in a patient with degenerative mitral regurgitation Perfusion 2024 39 06 1277 1279 37354131 10.1177/02676591231186725

[4] Shibasaki I Otani N Saito S Overview of mechanical circulatory support for the management of post-myocardial infarction ventricular septal rupture J Cardiol 2023 81 05 491 497 36503063 10.1016/j.jjcc.2022.12.001

[5] Clements S D Jr Story W E Hurst J W Craver J M Jones E L Ruptured papillary muscle, a complication of myocardial infarction: clinical presentation, diagnosis, and treatment Clin Cardiol 1985 8 02 93 103 3971608 10.1002/clc.4960080206

[6] Kawanami S Egami Y Nishino M Tanouchi J One-week Impella CP support for papillary muscle rupture as a bridge to surgery: a case report Eur Heart J Case Rep 2023 7 07 ytad274 37501710 10.1093/ehjcr/ytad274 PMC10371041

[7] Elhussein T A Hutchison S J Acute mitral regurgitation: unforeseen new complication of the Impella LP 5.0 ventricular assist device and review of literature Heart Lung Circ 2014 23 03 e100 e104 24296306 10.1016/j.hlc.2013.10.098

[8] Meani P Lorusso R Pappalardo F ECPella: concept, physiology and clinical applications J Cardiothorac Vasc Anesth 2022 36 02 557 566 33642170 10.1053/j.jvca.2021.01.056

[9] Hori T Iida M Uchiyama M Shimokawa T Successful cases of percutaneous left ventricular assist device “Impella” to fulminant myocarditis J Cardiothorac Surg 2022 17 01 72 35414115 10.1186/s13019-022-01821-x PMC9004069

